# Closing the dietary fibre gap - developing a novel dietary fibre screening tool (SCREEN-IT) for the UK population: validity, reproducibility and usability insights

**DOI:** 10.1007/s00394-026-04033-4

**Published:** 2026-07-04

**Authors:** Victoria Norton, Julie A. Lovegrove, Michelle Weech, Eve F.A. Kelly, Stella Lignou

**Affiliations:** 1 Sensory Science Centre, Department of Food and Nutritional Sciences, Harry Nursten Building, University of Reading, Whiteknights, Reading, RG6 6DZ UK; 2 Hugh Sinclair Unit of Human Nutrition, Department of Food and Nutritional Sciences, Harry Nursten Building, University of Reading, Whiteknights, Reading, RG6 6DZ UK

**Keywords:** dietary fibre, screening tool, food frequency questionnaire, validity, usability, reproducibility

## Abstract

**Purpose:**

Insufficient dietary fibre intake can increase chronic disease risk with identification of effective strategies considered a public health priority. However, there are no UK-based dietary fibre intake tools to estimate consumption habits. This study addresses this gap by developing a novel dietary fibre screening tool (SCREEN-IT) for the UK population and explores validity, reproducibility and usability insights.

**Methods:**

SCREEN-IT was developed based on key dietary fibre-rich food categories in the UK diet. The validity of SCREEN-IT (10-item) was tested against a UK-based Food Frequency Questionnaire (eNutri-FFQ; 157-item) seven-days apart (*n* = 70; 55.1 ± 18.7 years). SCREEN-IT reproducibility (*n* = 155; 51.7 ± 18.2 years) was evaluated on two occasions (four-weeks apart) and usability (System Usability Scale; SUS) was assessed. Intra-class correlation coefficients, percentage difference, quartile agreement, weighted kappa and Bland-Altman plots were used to quantify agreement and extent of bias.

**Results:**

Agreement between methods (SCREEN-IT vs. eNutri-FFQ) was “acceptable to good agreement”; higher dietary fibre estimates from eNutri-FFQ (mean bias: −3.91 g/d). SCREEN-IT was quick to complete (< 5-min) with higher SUS than eNutri-FFQ (83.4 vs. 76.8/100). SCREEN-IT was reproducible on re-test (“acceptable to good agreement”), mean bias close to zero (−0.04 g/d), high usability (84.9/100) and received positive feedback (easy-to-use, functional, thought-provoking, enjoyable).

**Conclusion:**

SCREEN-IT was successfully developed with favourable validity, reproducibility and usability feedback. It was considered a suitable tool to estimate dietary fibre intake for the UK population. This novel tool could help raise dietary fibre awareness by promoting relevant food sources in a quick and easy way to increase future intake.

**Supplementary Information:**

The online version contains supplementary material available at https://doi.org/10.1007/s00394-026-04033-4.

## Introduction

Adherence to “healthy eating” practices (as defined by the nine food-based Eatwell Guide recommendations) is less than 0.1% in the UK population [1]. More specifically, dietary fibre has the lowest population-level compliance with dietary recommendations compared with other food groups (e.g., oily fish, sugar, non-oily fish, fruits and vegetables, saturated fat, red and processed meat, salt and total fat) [1]. This is supported by the recent National Diet and Nutrition Survey (NDNS) data where 96% of UK adults do not meet the 30 g/d dietary fibre recommendations proposed by the Scientific Advisory Committee on Nutrition (SACN) in 2015 [2, 3]. Dietary fibre is defined by SACN as “*all carbohydrates that are neither digested nor absorbed in the small intestine and have a degree of polymerisation of three or more monomeric units*,* plus lignin*” and includes a variety of plant-based sources (e.g., fruits, vegetables, nuts, seeds, pulses, wholegrains-rich foods) (hereafter referred to as ‘dietary fibre’) [3, 4]. Insufficient dietary fibre intake can lead to negative outcomes such as increased risk of cardiovascular disease mortality, pancreatic cancer and diverticular disease [5]. Accordingly, addressing the widespread underconsumption of dietary fibre in the UK population is an urgent priority to benefit public health.

Exploring the common underlying reasons for sub-optimal dietary fibre intake is fundamental to the development of future targeted strategies to shift consumption patterns. A variety of dietary fibre-derived challenges have been proposed including: (i) poor uptake of dietary fibre-rich foods (e.g., fruits, vegetables, pulses, wholegrains, nuts, seeds, etc.); (ii) lack of dietary fibre knowledge and awareness (health benefits, food sources, recommendations); (iii) communication (no mandatory labelling on food packaging); (iv) negative perceptions (side effects, sensorial properties); and (v) environment (availability, cost, food insecurity) [6–15]. Food intake (at an individual level) is rarely measured; therefore, it is a key issue to address and can contribute to dietary fibre misconceptions (such as mismatch between actual vs. perceived intake) [14–17]. However, common dietary assessment tools (e.g., food frequency questionnaires (FFQ), weighed food records, 24-hr recalls, etc.) tend to be time-consuming, require nutrition skills to interpret data as well as being prone to misclassification, measurement error and bias (recall, response, social desirability) [18–20]. Therefore, developing a more practical, user-friendly and quick screening tool to measure dietary fibre intake with application for different audiences could be fundamental.

Short dietary screening tools (<35-item) are a rapid strategy to quantify low or high intakes of particular foods and/or nutrients; therefore, they can highlight potential components for diet-derived change [21]. It is key that such tools are easy-to-use across varying levels of nutrition-based knowledge and in a range of settings [21]. To date, dietary fibre related tools have either been combined with measuring other nutrients (such as fat) or developed for New Zealand and Dutch populations [22–28]. Currently, there are no UK-based dietary fibre screening tools to assess or rank intake; accordingly, this research will address this gap by developing a quick and easy screening tool (SCREEN-IT) to quantify dietary fibre intake by adhering to guidelines for evaluating new dietary assessment tools [29]. More specifically, this study has three key objectives to: (1) create the dietary fibre screening tool (SCREEN-IT) for the UK population; (2) investigate validity of SCREEN-IT against a comprehensive, validated UK-based FFQ; and (3) test reproducibility and usability of SCREEN-IT balanced across three age-groups for representativeness (18–40, 41–64 and 65 + years).

## Methods

### Study design

**Fig. 1:**
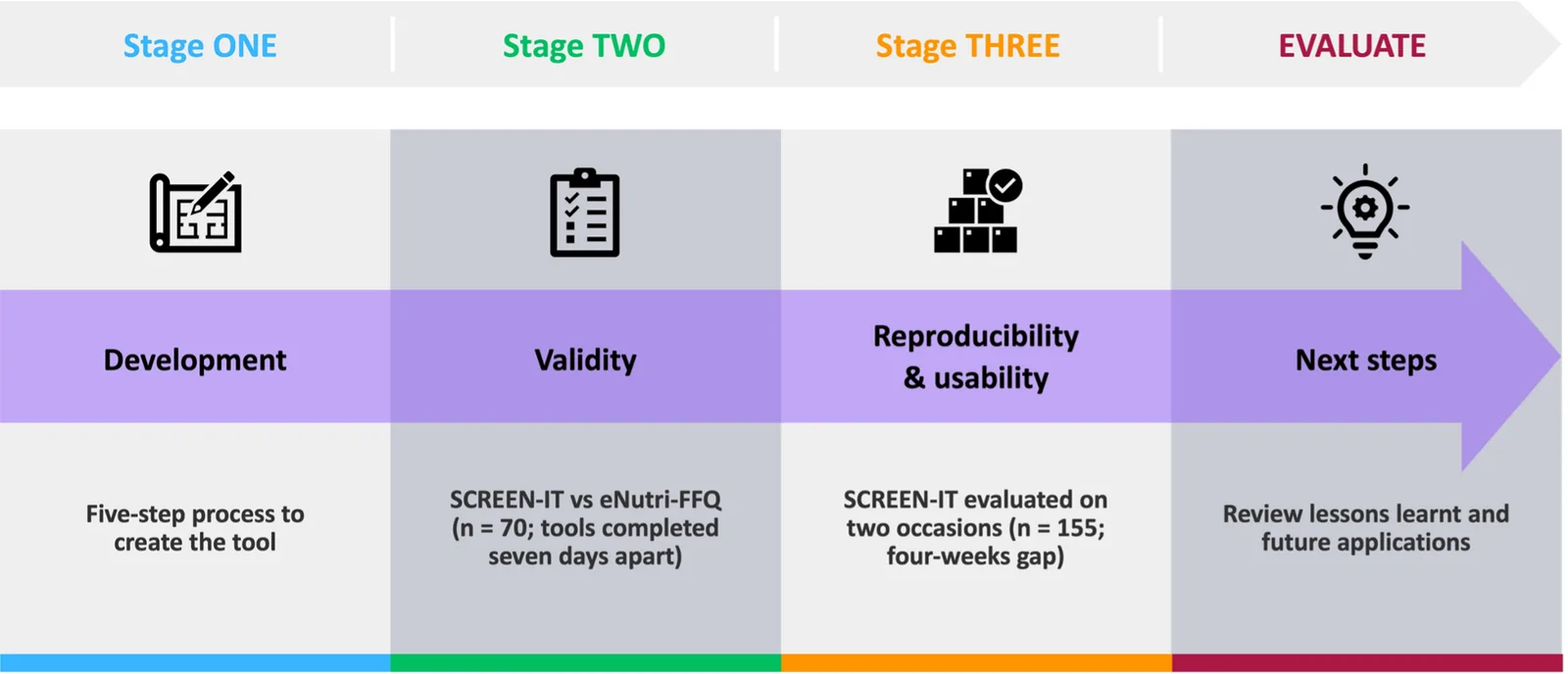
Overview of SCREEN-IT from development (stage one) to testing (validity, reproducibility, usability: stage two + stage three)

Fig 1 summarises the multi-stage approach used to develop and test SCREEN-IT. Participants aged 18 years and over were recruited (using word of mouth, departmental databases, community groups, posters distributed in the local area) in line with the inclusion criteria (no health conditions impacting food intake, not following a weight-loss diet, not pregnant or breastfeeding, no cognitive impairments, food allergies or intolerances, living in the UK and having access to a digital device). If interested, participants read the information sheet (described as a study on testing different communication strategies to prevent bias), had an opportunity to ask any questions and provided informed consent. The research received a favourable ethical opinion for conduct by the University of Reading School of Chemistry, Food and Pharmacy Research Ethics Committee (study number: 26/2024) and was performed in accordance with the Declaration of Helsinki as well as registered with clinicaltrials.gov (NCT06675630).

### Development of SCREEN-IT

**Fig. 2:**
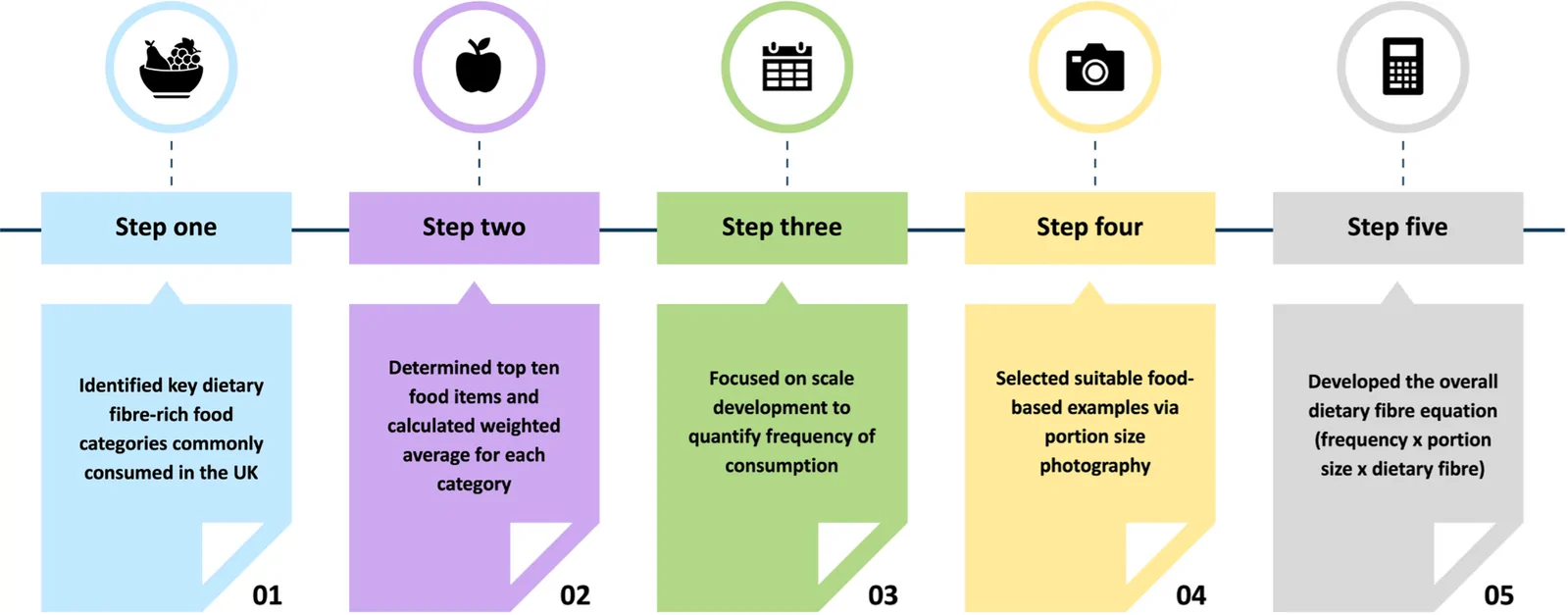
Summary of the five-step development process of SCREEN-IT

SCREEN-IT development was a five-step process as summarised in Fig. 2 with the overall goal of creating a quick and easy tool to capture dietary fibre intake in the UK population.

**Step one**: identification of key dietary fibre-rich food categories was based on commonly consumed foods within the UK diet and/or likely to resonate (user-friendly language) to help develop the tool with food-level insights captured from the NDNS [2]. This was supported by: (a) a previous Dutch dietary fibre-related screening tool for design purposes; (b) analysis of commonly consumed dietary fibre-rich foods (all selected foods groups were within this ranking apart from vegetables); and (c) dietary fibre categories were initially piloted for suitability in a real-world setting and based on this feedback an extra category (dietary fibre snacks) was included to capture food items such as cereal bars, oatcakes, flapjack and popcorn [28, 30, 31]. This resulted in selection of ten dietary fibre-rich food groups: (1) wholemeal bread; (2) dietary fibre-rich cereals; (3) wholemeal pasta and rice; (4) potatoes; (5) dried fruits; (6) fresh and frozen fruits; (7) vegetables; (8) pulses; (9) nuts and seeds; and (10) dietary fibre-rich snacks (Table S1).

**Step two**: determination of the ten most frequently consumed food items within each category were determinated utilising the NDNS food-level data (Rolling Programme Years 6–9) to enable direct comparison with the selected UK-based FFQ [32]. The dietary fibre content (AOAC) per 100 g was collated from the McCance and Widdowson’s Composition of Foods Integrated Dataset [33] to calculate a weighted mean dietary fibre content per food item and category based on frequency of consumption.

**Step three**: focused on scale development; a six-point scale (never to always) was used to capture consumption habits during the previous two weeks [28]. The resulting daily frequencies were defined quantitively as never (no; 0), rarely (less than once; 0.14), sometimes (one to two days; 0.29), often (three to four days; 0.57), mostly (five to six days; 0.86) to always (daily; 1) [28].

**Step four**: selected two representative food-based examples per dietary fibre-rich category to support portion size estimation derived from healthy eating guidance and this determined dietary fibre (g) per typical portion size [34]. For example, 80 g of baked beans and chickpeas were presented for pulses (Figure S1) [35, 36]. The selected foods (*n* = 20) were prepared, cooked (as required), weighed and plated for this purpose. A series of digital photographs were taken using an agreed protocol by a professional photographer (Bennetto Photography, Reading, UK) with previous food-based photography experience [32, 37, 38]. The number of portions was only visible for SCREEN-IT if a frequency between “rarely to always” was selected. Five options were presented from “one portion to five or more portions” (with a comment box if outside this range) where this information was then defined quantitively (e.g., one portion is one and so on).

**Step five**: developed the dietary fibre calculation adapted from Rijnaarts et al. [28]: frequency [six-point scale] x portion size [five-point scale] x dietary fibre content [g per typical portion size]; all ten dietary fibre categories were then aggregated to provide an overall dietary fibre intake (g/d) (Figure S2).

### SCREEN-IT validity

This stage estimated dietary fibre intake from SCREEN-IT compared with a UK based eNutri-FFQ in line with previous research (country-specific FFQ and two-week timeframe) [27, 28]. Cade et al. [39] noted that a mimimum of 50 participants was a suitable sample size for FFQ validation studies. To cater for potential dropouts or implausible data, at least 77 participants were invited to complete SCREEN-IT or eNutri-FFQ and then seven days later fill out eNutri-FFQ or SCREEN-IT using a single-blinded randomised crossover design.

SCREEN-IT was self-administered and deployed digitally using the Compusense platform (Compusense Cloud, Ontario, Canada). Participants were presented with ten dietary fibre-rich categories monadically and asked if they consumed each category in the previous two weeks (six-point scale: never to always). If so, the number of portions typically consumed were recorded (five-point scale: one to five or more portions with a comment box option if outside range) supplemented with two portion size photographs per category. The overall dietary fibre value (g/d) was manually calculated using the equation described in step five. eNutri-FFQ is a web-based tool that quantifies food and nutrient intake from the previous four weeks via a validated, up-to-date and comprehensive UK-based FFQ as described elsewhere [32, 40, 41]. Prior validation of eNutri-FFQ demonstrated acceptable agreement of dietary fibre intakes against three-day weighed diet records [32]. In brief, it records how often participants consumed each of the 157-items [food+drink] (not in the last month, less than once a week, once a week, 2–4 times a week, 5–6 times a week, once a day, twice a day, 3–4 times a day or 5 + times a day) and usual portion sizes via selecting one of seven portion size buttons (three photographs and four text descriptions). Using the inputted data, eNutri automatically calculates the mean daily intakes of food items and subsequently estimates intakes of a range of food groups and nutrients.

For both tools, three key parameters were recorded: dietary fibre intake (g/d), time to complete (min; from software derived timestamps) and usability (10-item System Usability Scale (SUS) as outlined by Brooke [42] with a five-point scale (strongly disagree to strongly agree) and converted into a score out of 100). In addition, basic demographic information was captured (age, sex, self-reported weight/height, living status, education level and employment type).

### SCREEN-IT reproducibility and usability

This stage focused on assessing tool reproducibility and usability using a larger sample size balanced across three key age-groups (younger: 18–40: middle: 41–64 and older: 65 + years) for representativeness aiming for 50 participants per group [39]. Participants (*n* = 165 to allow for 10% dropouts) were invited to complete SCREEN-IT on two occasions four-weeks apart (retest approach; SCREEN-IT 1 and SCREEN-IT 2) on the Compusense platform. Five parameters were recorded (i) usability (SUS) [42]; (ii) device screen size (small: smartphone; medium: tablets; and large: laptop/desktop computer); (iii) time to complete from software derived timestamps (min); (iv) feedback on SCREEN-IT (open-ended comment box); and (v) basic demographic information.

### Statistical analysis

A pre-defined statistical protocol was utilised in line with recommendations for testing validity of dietary assessment tools and interpretation criteria as described by Lombard et al. [29]. Dietary fibre intake (g/d) was the variable of interest for comparison using the following techniques: (1) intra-class correlation coefficients (ICC) agreement between tools (poor: <0.20, acceptable: 0.20–0.59, good: >0.61) using the inter-rater model; (2) paired samples t-test (agreement at group level); (3) percentage difference (size and direction of error) where the mean difference (A – B) was divided by average of both methods ((A + B)/2) multiplied by 100 (poor: >20.0%; acceptable: 11.0–20.0%; good: 0.0-10.9%); (4) cross-classification (agreement at an individual level) was estimated via quartile analyses to calculate the proportion of participants within exact (same quartile for both methods), adjacent (one quartile apart) or extreme (opposite quartiles) agreement; (5) Cohen’s weighted kappa agreement at an individual level (poor: <0.20; acceptable: 0.20–0.59; good: >0.61) was performed on categorical data computed utilising a contingency table; and (6) Bland-Altman plots (presence, direction and extent of bias) were visualised in accordance with Bland and Altman methodology [43] in XLSTAT (version 2023.3.1.1416, New York, USA) and GraphPad Prism (version 10.4.2 (534), Boston, USA). In addition, age-related differences were explored using analysis of variance (ANOVA) and if significant, Tukey’s Honestly Significant Difference test for pairwise comparisons; *p* < 0.05 was considered significant for relevant analyses. Feedback on SCREEN-IT was captured by an open-ended question and collated by identifying recurring themes (e.g., at least five mentions) [44].

## Results

### Participant demographics

Four participants in the validity assessment reported daily energy intake from eNutri-FFQ which was considered implausible in accordance with non-individualised cut-offs advised by Willett (female: 500–3500 kcal/d and male: 800–4000 kcal/d) [45]. Three participants did not complete both dietary assessments within the agreed timeframe; hence, such data was excluded from subsequent analyses (Figure S3). Seventy participants (55.1 ± 18.7 years) were included in the validity analyses as summarised in Table 1. Most participants were within the healthy body mass index (BMI) range, living with a partner, had a university-based education and were either employed or retired. Overall, 155 participants (51.7 ± 18.2 years) were included in the reproducibility and usability assessment (six and four participants did not complete both assessments or were outside agreed timeframe, respectively) consisted of balanced numbers across the three age groups with similar demographics to the validity stage (Table 1).

**Table 1:** Demographic summary for validity (SCREEN-IT vs. eNutri-FFQ; *n* = 70) and reproducibility [+ usability] (SCREEN-IT 1 vs. SCREEN-IT 2; *n* = 155)

Demographic	Validity		Reproducibility	
	*n*	%	*n*	%
*Age (years)*				
18–40	23	32.9	52	33.5
41–64	16	22.9	52	33.5
65+	31	44.3	51	32.9
*Sex*				
Male	35	50.0	70	45.2
Female	35	50.0	85	54.8
*BMI (kg/m* ^*2*^ )*				
Underweight (< 18.5)	1	1.4	3	1.9
Healthy range (18.5–24.9)	39	55.7	76	49.0
Overweight + obese (> 25)	30	42.9	76	49.0
*Living status*				
Live alone	8	11.4	27	17.4
Live with partner	37	52.9	73	47.1
Live with family	21	30.0	49	31.6
Live in shared accommodation	4	5.7	6	3.9
*Education level*				
Doctorate degree	5	7.1	16	10.3
Postgraduate degree	22	31.4	42	27.1
Undergraduate degree	18	25.7	44	28.4
Vocational/technical training	15	21.4	28	18.1
Secondary school	10	14.3	24	15.5
Primary school	0	0	1	0.6
*Employment type*				
Employed (full-time)	21	30.0	52	33.5
Employed (part-time)	8	11.4	22	14.2
Freelancer/contractor	1	1.4	6	3.9
Self-employed	5	7.1	6	3.9
Unemployed	2	2.9	6	3.9
Homemaker	1	1.4	4	2.6
Student	2	2.9	12	7.7
Retired	30	42.9	47	30.3

*BMI (body mass index) calculated from self-reported body weight and height.

### SCREEN-IT validity

**Fig. 3:**
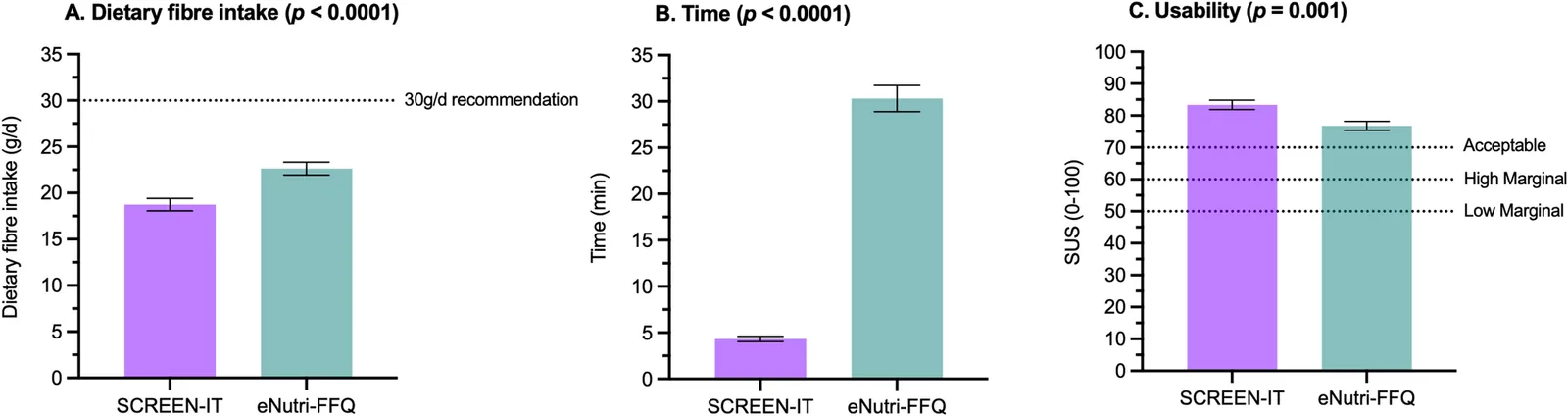
Validity metrics (*n* = 70; SCREEN-IT vs. eNutri-FFQ) by **A** estimated dietary fibre intake (g/d); **B** completion time (min); and **C** usability of tools (System Usability Scale; SUS). Data reported as mean ± standard error

**Fig. 4:**
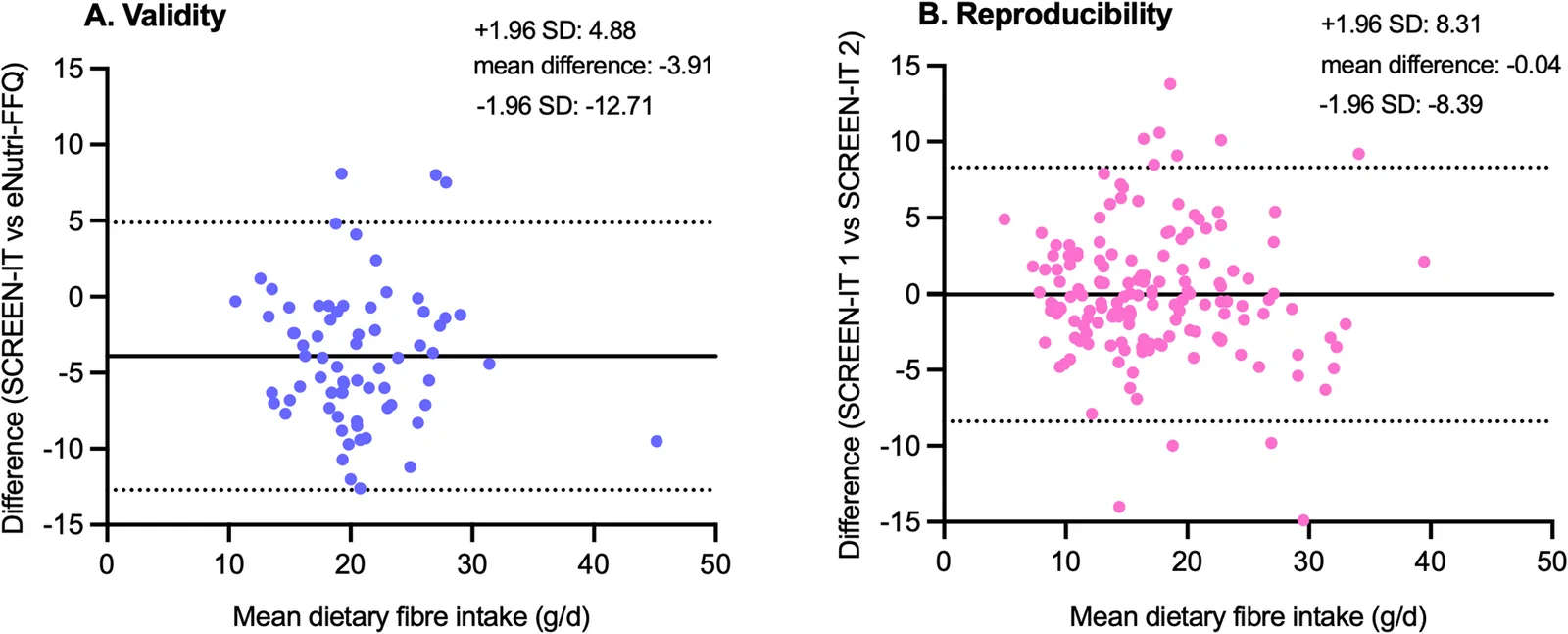
Bland-Altman plots (mean difference – black line and limits of agreement – dotted lines) for estimated dietary fibre intake (g/d) for validity (SCREEN-IT vs. eNutri-FFQ; *n* = 70) and reproducibility (SCREEN-IT 1 vs. SCREEN-IT 2; *n* = 155)

Mean estimated dietary fibre intake was significantly lower (3.9 g/d) using SCREEN-IT compared with eNutri-FFQ (Fig. 3; *p* < 0.0001). The agreement was classified as “acceptable to good” despite differences between methods for ICC, percentage difference, quartile agreement and weighted kappa (Table 2). The exact and extreme quartile agreement were identified as 52.9% and 10.0% of the data, respectively. The Bland-Altman plot (Fig. 4) highlighted that 5.7% of the data points were outside the limits of agreement (-12.71 to 4.88 g/d) and mean bias (-3.91 g/d) demonstrated higher dietary fibre estimates for eNutri-FFQ than SCREEN-IT. SCREEN-IT was significantly quicker to complete (by 26-min); however, both tools had acceptable usability with higher scores (6.6 points) cited for SCREEN-IT compared with eNutri-FFQ (Fig. 3; *p* < 0.05).

**Table 2:** Summary of statistics with relevant interpretation for validity (*n* = 70: SCREEN-IT vs. eNutri-FFQ) and reproducibility (*n* = 155: SCREEN-IT 1 vs. SCREEN-IT 2)

Dietary fibre (g/d)	ICC^1^	% difference^2^	Quartile agreement^3^	Weighted kappa^4^
Validity	0.72	18.9	52.9	0.37
Interpretation*	Good	Acceptable	Good	Acceptable
Reproducibility	0.89	0.27	60.6	0.48
Interpretation*	Good	Good	Good	Acceptable

Age-derived findings are reported in Table S2; *interpretation criteria as described by Lombard et al. [29]:

^1^Intra-class correlation coefficient (ICC) [good: >0.61, acceptable: 0.20–0.59, poor: <0.20]

^2^% difference [good: 0.0-10.9%, acceptable: 11.0–20.0%, poor: >20.0%]

^3^Cross-classification [good: >50%, poor: <50%; validity- exact: 52.9%, adjacent: 37.1%, extreme: 10.0% and reproducibility - exact: 60.6%, adjacent: 31.6%, extreme: 7.7%]

^4^Weighted kappa [good: >0.61, acceptable: 0.20–0.59, poor: <0.20]

### SCREEN-IT reproducibility and usability

Overall, there were no significant differences in mean dietary fibre intake (between SCREEN-IT 1 and SCREEN-IT 2 [17.2 ± 0.5 vs. 17.2 ± 0.6 g/d]); 3.2% and 6.5% meeting the 30 g/d recommendation, respectively. It was demonstrated that SCREEN-IT was reproducible with either “acceptable or good agreement” for ICC, percentage difference, quartile agreement and weighted kappa (Table 2). Fig 4 illustrated that 7.1% of the data points were outside the limits of agreement (-8.39 to 8.31 g/d); however, the mean bias was close to zero (-0.04 g/d). There was a tendency for SCREEN-IT to be completed marginally faster the second time (3.4 vs. 3.8 min; *p* = 0.06).

**Fig. 5:**
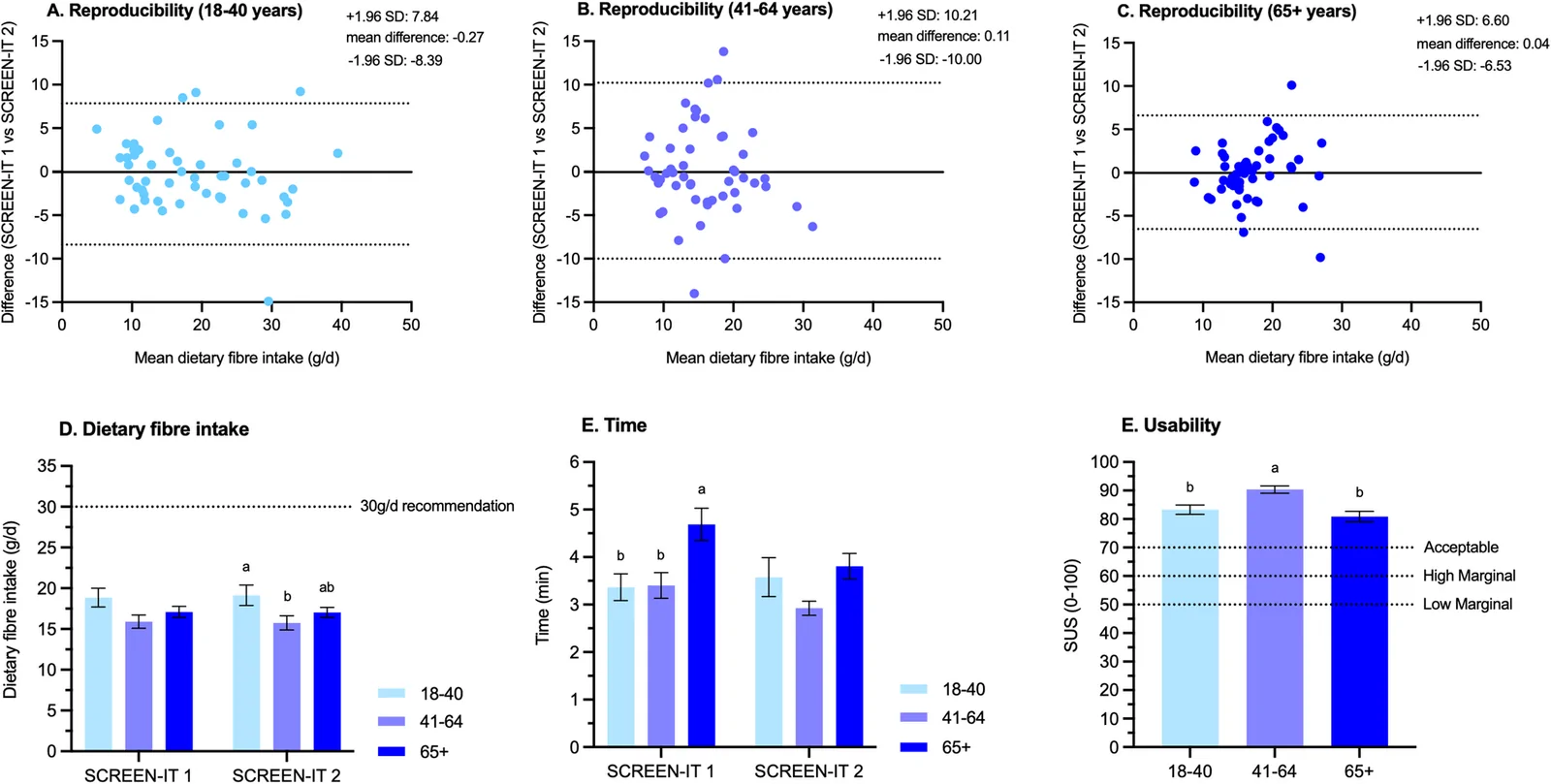
Age-related metrics from SCREEN-IT 1 and SCREEN-IT 2 (*n* = 155; 18–40 [*n* = 52]; 41–64 [*n* = 52]; and 65+ [*n* = 51]) by **A–C** Bland-Altman plots (mean difference – black line and limits of agreement – dotted lines) for estimated dietary fibre intake (g/d); **D** estimated dietary fibre intake (g/d); **E** SCREEN-IT completion time; and **F** SCREEN-IT usability (System Usability Scale; SUS) by age groups. Data reported as mean ± standard error and differing letters reflect significance from pairwise comparisons

The youngest cohort had higher mean dietary fibre intakes compared with older cohorts post SCREEN-IT 2 (*p* = 0.04); however, no age-related differences were reported for SCREEN-IT 1 (Fig. 5). Age-derived reproducibility was either “acceptable or good in agreement” for ICC, percentage difference, quartile agreement and weighted kappa (apart from 18 to 40 years quartile agreement was perceived as poor) (Table S2). Between 5.8 and 7.7% data points were outside the limits of agreement for each age-group but mean bias was close to zero (Fig. 5). Older adults spent more time completing SCREEN-IT 1 compared with younger cohorts (3.4 vs. 4.7 min; *p* = 0.002). This trend continued at SCREEN-IT 2; however, there was no significant difference (*p* = 0.10). The SUS values indicated acceptable usability for all age-groups (84.9/100); however, the middle-aged cohort (41–64 years) perceived the tool more usable compared with the other age groups (Fig. 5; *p* < 0.0001).

SCREEN-IT was completed using different sized devices: smartphones (small; 31.6%), tablets (medium; 7.7%) and laptops/desktop computers (large: 60.6%) with no significant differences in perceived tool usability between devices (*p* = 0.90).

The feedback on SCREEN-IT was very positive (81.4% of comments) with key themes emerging such as **easy-to-use** *“all well laid out*,* very easy to understand*,* examples of foods are very nice*,* means I know how to answer”*, **functional** *“I think it is the best tool that I have ever used. I have learnt a lot from it so far and I have liked the idea of changing my meal diet”*, **thought-provoking** *“it’s a very useful tool for making you actually count how much fruit*,* veg etc. you are actually eating vs. how much you think you are eating”*, **enjoyable** “*I liked the tool. It’s easy to use*,* provides good explanations and was fun (never thought I would say a questionnaire could be fun!!!)”* and **concise** “*quick and easy. Two-week time period easier to recall than one month*”. The negative comments (less than 20% of comments) were centred on two areas: **portion size estimation** “*the portion samples aren’t easy to translate to what goes on the plate each day (it’s not weighed out before each serving! )”* and **more agile approach** “*I sometimes find the portion comparisons a bit limiting for me”.*

## Discussion

The novel SCREEN-IT tool was successfully developed adopting a multi-stage process utilising insights from NDNS, previous dietary fibre-derived research and food-based examples with relevant portion size photographs to ensure suitability and relevance for a UK population. Key objectives were tool development, initial validation (against FFQ) and reproducibility (re-test on two occasions) to understand areas for improvement and future applications of SCREEN-IT.

Validity was conducted in line with previous research and best practices; for example, SCREEN-IT was compared with a country-specific FFQ (one of the most common methods to capture habitual food intake) using a relevant sample size (*n* = 70) for this purpose [27–29, 39]. The estimated dietary fibre intake from SCREEN-IT was lower compared with eNutri-FFQ as expected. SCREEN-IT included considerably fewer food items (10 vs. 157-item) and category groupings (e.g., nuts, seeds, snacks, etc.). This may have contributed to reduced data granularity; therefore, limiting dietary fibre quantification from all food sources [27, 28]. The differing timeframes of dietary assessment (two weeks [SCREEN-IT] vs. four weeks [eNutri-FFQ]) may also have contributed to the observed differences. Rijnaarts et al. [28] validated a dietary fibre screening tool for a Dutch population focusing on key dietary fibre food categories (fruit, vegetables, whole-grain products, pasta, rice, potatoes, legumes, nuts and seeds). They also reported their dietary fibre screening tool had lower recorded intakes compared with a more comprehensive FFQ [28]. Similar findings were present in a short food frequency questionnaire (DFI-FFQ) to identify low, medium and high dietary fibre intakes in New Zealand; however, this research was limited by the number of dietary fibre-rich food categories (vegetables, fruits, breads + cereals, nuts + seeds, legumes) [27]. The overall agreement between methods (SCREEN-IT vs. eNutri-FFQ) was categorised as “acceptable to good” suggesting SCREEN-IT was a suitable tool for quantifying and/or ranking dietary fibre intake in the UK. In addition, despite the relatively low dietary fibre intake per se, it was possible to rank participants based on low or high intake. This is increasingly relevant since only 4.0% of the UK population meet dietary fibre recommendations [2]; therefore, this tool is warranted and could highlight gaps in consumption habits to drive future strategies to increase intake. Moreover, the good usability and speed of completion (less than 5-min) demonstrated its suitability for use in different settings (e.g., home, healthcare, communities, supermarkets, etc.) and its potential role as an educational resource in contexts where nutrition expertise can be limited. Further insights beyond initial validity with a FFQ could include prospective dietary assessment methodologies (weighed food records, 24-hr recalls) and time-periods (short vs. long-term) to understand variation as well as additional analyses by dietary fibre-rich categories. Objective metrics are an important aspect of validating dietary assessment tools but identifying relevant biomarker(s) for total dietary fibre (rather than specific dietary fibre foods) is a cited challenge within the literature [46].

The reproducibility and usability assessment included a larger cohort to enable insights from three distinct age-groups. Utilising a life-course approach can help identify “populations of interest” and opportunities for change, as well as understanding any unique challenges associated with each age-group [47, 48]. SCREEN-IT was reproducible (“acceptable or good”) based on capturing dietary fibre-related trends from two occasions (four-weeks apart) using a range of statistical agreement-based techniques and this was broadly similar across all three age-groups. Key metrics such as time, usability and device size were also recorded. Initially, older adults took more time to complete SCREEN-IT compared with both younger age-groups (main age-related difference). However, completion time was quicker for SCREEN-IT on the second occasion suggesting exposure and familiarity with such a tool was beneficial. Usability scores (SUS) were comparable across all three age-groups; previously such scores from dietary assessment tools were lower in older adults compared with other age-groups [34]. SCREEN-IT usability was high (84.9/100); therefore, similar to common everyday technologies (Gmail: 83.5, microwaves: 86.9) and current UK dietary assessment tools (Intake24: 71.5, eNutri-FFQ: 74.0) [32, 49, 50]. It is important that the tool was evaluated using different device sizes (small to large) to reflect common societal usage (smartphone, tablet, PC and laptop; [51]) and ensure suitability as well as providing future design cues. Feedback was positive and key themes included: easy-to-use, functional, thought-provoking, enjoyable and concise. This demonstrates SCREEN-IT’s potential as an educational resource to raise awareness compared with typical dietary assessments. One of the challenges raised with the SCREEN-IT tool was the portion size estimation to facilitate this process (despite two photographs presented per category); however, this is not unique to SCREEN-IT but appears to be ongoing issue for all dietary assessment methods [52]. Accordingly, adding more portion size photographs as examples and/or providing more detailed instructions could be beneficial; however, a balance is needed as more text and photographs would impact the simple design as well as time to complete (two key benefits of SCREEN-IT) [40]. In addition, there are challenges with contextualising guidance (e.g., what is a standard portion size and how to visualise?) resulting in difficulty translating Eatwell Guide information into everyday eating habits [53].

Short dietary screening tools (<35-items) are inherently unable to provide complete data on intake but can estimate intakes for specific nutrients and highlight dietary components for improvement [21]. This research adhered to best practice for validation of dietary assessment tools by evaluating agreement between methods at individual and group levels (including different statistical techniques, interpretation criteria, sample size) [29, 39]. The dietary fibre categories (*n* = 10) were informed from pilot testing and captured food-level insights from the NDNS; however, consideration of meat-based alternatives and other relevant sources (including supplements) could help improve dietary fibre estimates in the future. All dietary assessment tools are associated with key challenges such as bias (e.g., self-reported, reliance on memory, social desirability, etc.), misclassification and measurement error [16, 18–20]. This may contribute to the higher dietary fibre estimates from the FFQ and/or result in overreporting of certain categories such as fruit and vegetables. The rationale for the two-week timeframe was twofold to support a more user-friendly approach (e.g., minimise burden, recall challenges, etc.) and in line with previous research [28]; therefore, differences in assessment time period (SCREEN-IT vs. eNutri-FFQ) may explain some of the variation identified between methods. In addition, despite using a randomised cross-over design, order effects are common in dietary assessments, typically resulting in lower intake on second measurements without necessary translating into an actual change in food intake [32]. Following evaluation, four key areas for improvement and future research were identified as summarised in Table 3.

**Table 3:** Next steps for SCREEN-IT based on initial validity, reproducibility and usability insights

Development areas	Rationale
Stakeholder engagement	This will provide feedback on design cues for automated feedback by stakeholders interested in delivering, using and/or benefiting from SCREEN-IT to inform the new digital model.
Digital model with automated feedback	∙ Updating SCREEN-IT to enable automatic dietary fibre estimates will be very valuable for any user or researcher. This would support delivery of personalised feedback to encourage behavioural change. ∙ The end goal is an open-access webpage with paper and digital versions of SCREEN-IT. This will also help with: (i) adapting SCREEN-IT for use across Europe and beyond to evaluate cross-cultural differences as well as lessons learnt from other countries; and (ii) regularly updating nutritional data for latest iteration of NDNS food-level data.
Validate against different dietary assessment tools	SCREEN-IT was only compared against FFQ and other methods (such as 24-hr recalls or weighed food records) could help with additional validation and continual improvement.
More diverse and representative UK cohort	SCREEN-IT will be updated to include relevant context and/or more instructions – this was not explicitly explained to prevent overestimating dietary fibre. SCREEN-IT will also be evaluated with different subgroups (e.g., children, hard-to-reach groups, varying level of nutrition expertise, device types, etc.) to understand scalability for public health benefit.

## Conclusion

The novel SCREEN-IT tool was successfully developed as a quick and easy approach to capture dietary fibre intake with favourable initial validity, reproducibility and usability feedback. The validity stage focused on a comparison with a country-specific FFQ highlighting reasonable agreement between methods; key advantages were quick to complete and easy-to-use. The reproducibility aspect demonstrated SCREEN-IT’s suitability and usability across the adult life-course. The positive feedback will inform future research to enable continual tool development. SCREEN-IT could also help to promote dietary fibre intake; thereby, improving adherence to 30 g/d recommendations by highlighting relevant food sources resulting in noteworthy public health benefits. The potential appeal of this tool is widespread with various options for implementation such as use in different settings (e.g., care-homes, healthcare, kitchens, supermarkets, communities), practical applications (including educational resources and toolkits) and/or characterisation of participants dietary fibre intake for intervention studies.

## Electronic Supplementary Material

Below is the link to the electronic supplementary material.

Supplementary Material 1

## Data Availability

Data described in this manuscript will be made available upon reasonable request from the corresponding author.

## Abbreviations

ANOVA: Analysis of variance
AOAC: Association of Official Analytical Chemists
BMI: Body mass index
FFQ: Food frequency questionnaire
ICC: Intra-class correlation coefficients
NDNS: National Diet and Nutrition Survey
SACN: Scientific Advisory Committee on Nutrition
SUS: System Usability Scale
