# Correction: Closing the dietary fibre gap - developing a novel dietary fibre screening tool (SCREEN-IT) for the UK population: validity, reproducibility and usability insights

**DOI:** 10.1007/s00394-026-04071-y

**Published:** 2026-09-25

**Authors:** Victoria Norton, Julie A. Lovegrove, Michelle Weech, Eve F. A. Kelly, Stella Lignou

**Affiliations:** 1 Sensory Science Centre, Department of Food and Nutritional Sciences, Harry Nursten Building, University of Reading, Whiteknights, Reading, RG6 6DZ UK; 2 Hugh Sinclair Unit of Human Nutrition, Department of Food and Nutritional Sciences, Harry Nursten Building, University of Reading, Whiteknights, Reading, RG6 6DZ UK

**Correction to: European Journal of Nutrition (2026) 65:198**

**https://doi.org/10.1007/s00394-026-04033-4**

In the original version of this article, figure 1 caption was mistakenly included at the end of the paragraph of the section ‘’Study design’’. The paragraph, which previously read as 

Fig 1 summarises the multi-stage approach used to develop and test SCREEN-IT. Participants aged 18 years and over were recruited (using word of mouth, departmental data¬bases, community groups, posters distributed in the local area) in line with the inclusion criteria (no health conditions impacting food intake, not following a weight-loss diet, not pregnant or breastfeeding, no cognitive impairments, food allergies or intolerances, living in the UK and having access to a digital device). If interested, participants read the infor¬mation sheet (described as a study on testing different com¬munication strategies to prevent bias), had an opportunity to ask any questions and provided informed consent. The research received a favourable ethical opinion for conduct by the University of Reading School of Chemistry, Food and Pharmacy Research Ethics Committee (study number: 26/2024) and was performed in accordance with the Dec¬laration of Helsinki as well as registered with clinicaltri¬als.gov (NCT06675630) Overview of SCREEN-IT from development (stage one) to testing (validity, reproducibility, usability: stage two + stage three).

but should have read as

Fig 1 summarises the multi-stage approach used to develop and test SCREEN-IT. Participants aged 18 years and over were recruited (using word of mouth, departmental data¬bases, community groups, posters distributed in the local area) in line with the inclusion criteria (no health conditions impacting food intake, not following a weight-loss diet, not pregnant or breastfeeding, no cognitive impairments, food allergies or intolerances, living in the UK and having access to a digital device). If interested, participants read the infor¬mation sheet (described as a study on testing different com¬munication strategies to prevent bias), had an opportunity to ask any questions and provided informed consent. The research received a favourable ethical opinion for conduct by the University of Reading School of Chemistry, Food and Pharmacy Research Ethics Committee (study number: 26/2024) and was performed in accordance with the Dec¬laration of Helsinki as well as registered with clinicaltri¬als.gov (NCT06675630).

The original article has been corrected.

