# Supplementary material for: Closing the dietary fibre gap - developing a novel dietary fibre screening tool (SCREEN-IT) for the UK population: validity, reproducibility and usability insights
Source: Eur J Nutr. 2026 Jul 4;65(5):198. doi: 10.1007/s00394-026-04033-4 (PMC13332889; doi:10.1007/s00394-026-04033-4)
Supplement: Supplementary file 1 — Supplementary Material 1 [file 394_2026_4033_MOESM1_ESM.docx]

**SUPPLEMENTARY**

**FREQUENCY [rate ten key dietary fibre food categories]**

**Table S1.** Did you **consume** any of the following **foods** in the **last two weeks**? Please select one response per food type.

| **Food Category** | **Examples** | **Never** | **Rarely** | **Sometimes** | **Often** | **Mostly** | **Always** |
| --- | --- | --- | --- | --- | --- | --- | --- |
| **Wholemeal bread**  **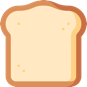** | Granary, 50:50, multi-seed, brown & wheatgerm bread/rolls |  |  |  |  |  |  |
| **Dietary fibre-rich cereals**  **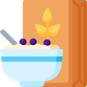** | Oats, porridge, bran flakes, Weetabix & muesli |  |  |  |  |  |  |
| **Wholegrain pasta + rice**  **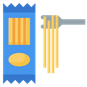** | Brown pasta/rice, wholemeal, quinoa & buckwheat |  |  |  |  |  |  |
| **Potatoes**  **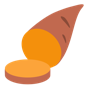** | Sweet, baked, boiled, mashed & new potatoes |  |  |  |  |  |  |
| **Dried fruits**  **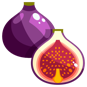** **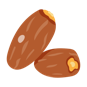** **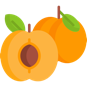** | Apricots, raisins, dates, prunes & cranberries |  |  |  |  |  |  |
| **Fruits (fresh + frozen)**  **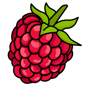** 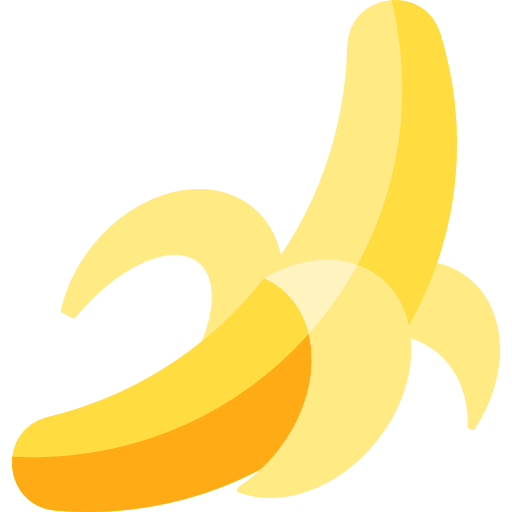 **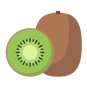** | Bananas, apples, grapes, blueberries & kiwi fruit |  |  |  |  |  |  |
| **Vegetables**  **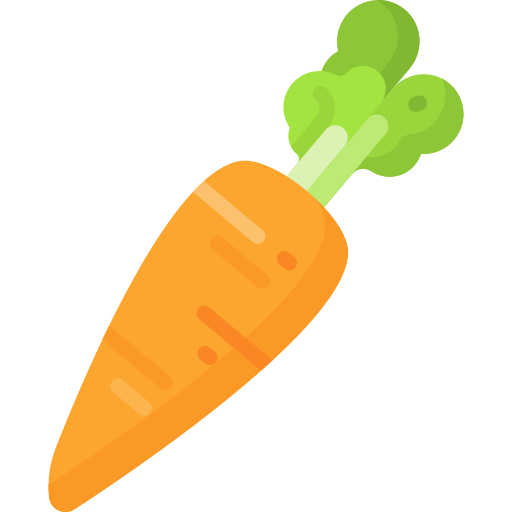** **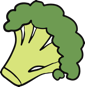** **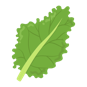** | Carrots, broccoli, kale, sweetcorn & Brussel sprouts |  |  |  |  |  |  |
| **Pulses**  **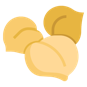** **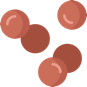** **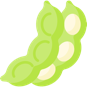** | Peas, lentils, chickpeas, baked beans & green beans |  |  |  |  |  |  |
| **Nuts + seeds**  **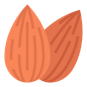** **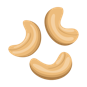** **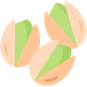** | Peanuts, almonds, walnuts, sunflower seeds & sesame seeds |  |  |  |  |  |  |
| **Dietary fibre-rich snacks**  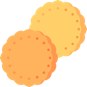 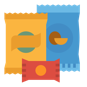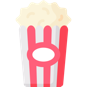 | Oatcakes, crackers, popcorn & flapjack |  |  |  |  |  |  |

**PORTION SIZE [ask a follow-up question if selected “rarely to always”]**

If so, what was your typical number of **PORTIONS**?

- - one portion
  - two portions
  - three portions
  - four portions
  - five or more portions
  - other (please specify): _________________


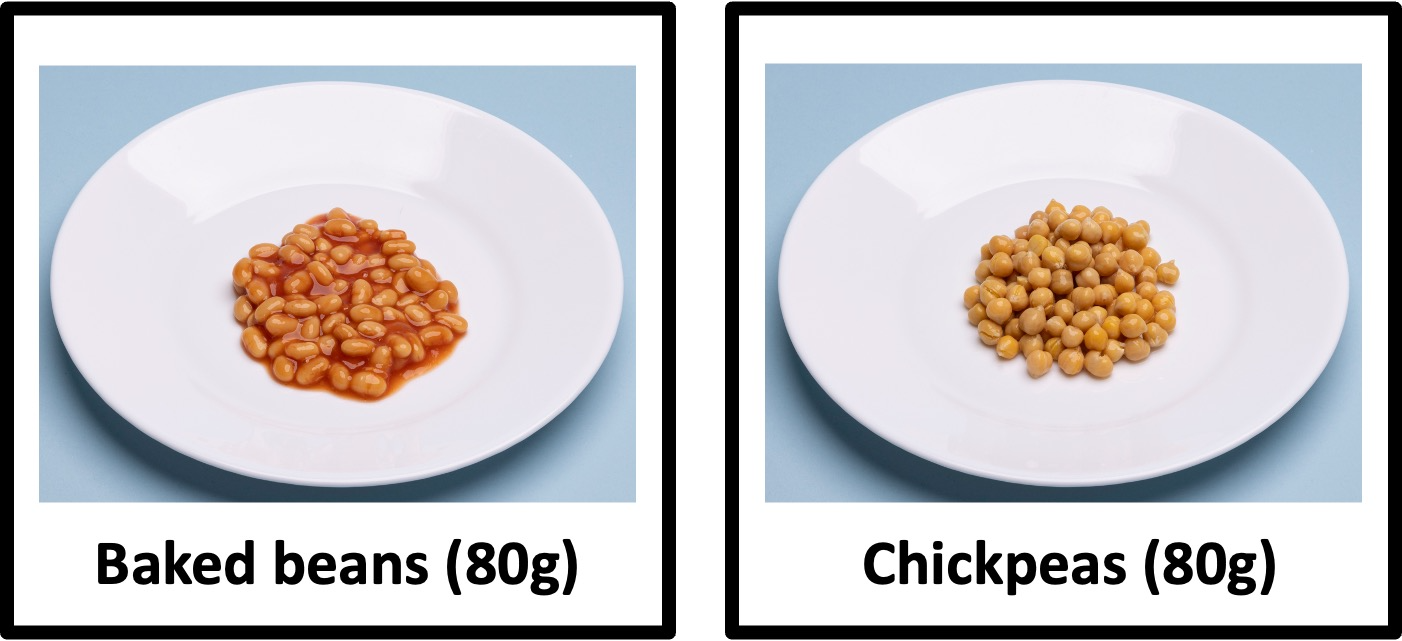


**Figure S1.** Example of supplied photographs (adhering to previous methodology [32,37,38]) for the pulse category.

**
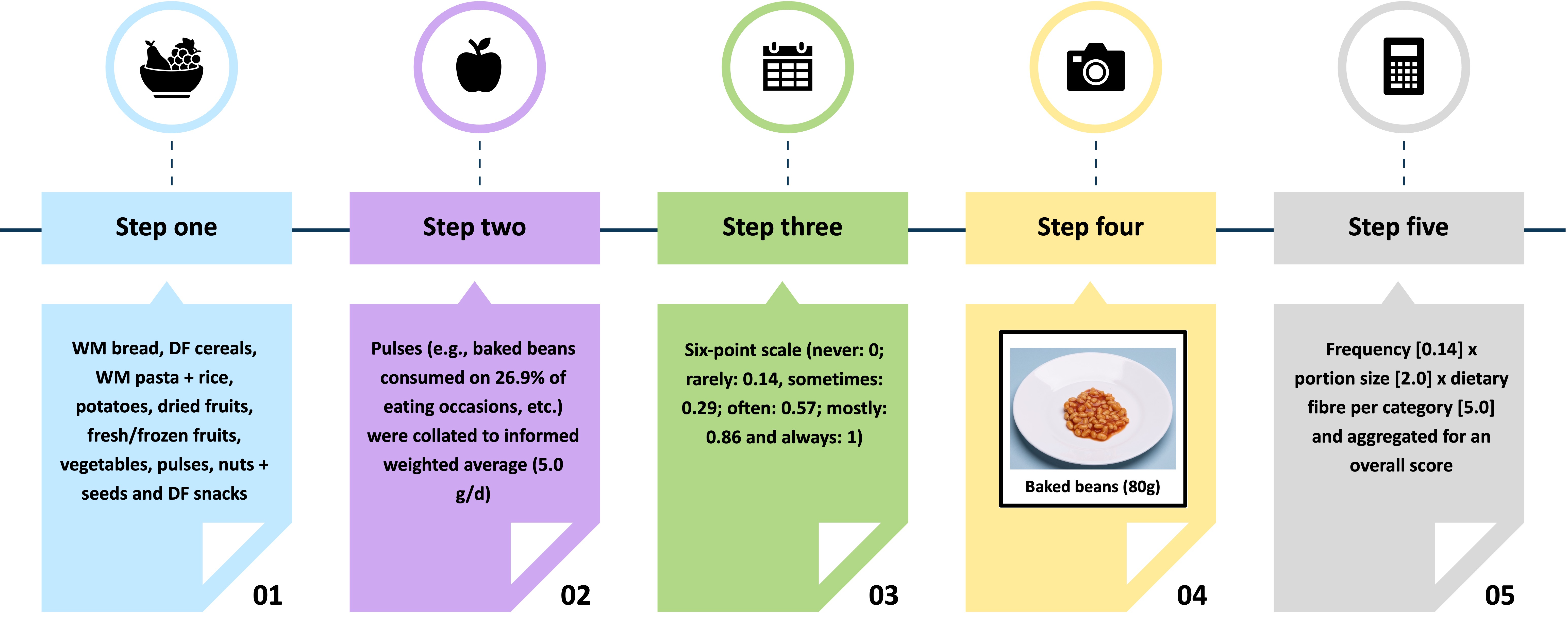
Figure S2.** Summary of the five-step process of SCREEN-IT with worked example from pulses. (1) step one: key dietary fibre-rich food categories (WM: wholemeal, DF: dietary fibre); (2) step two: determine top ten food items per category, record eating occasion and calculate weighted average); (3) step three: quantify frequency of consumption; (4) step four: two photographs per category (pulses: baked beans and chickpeas) with five-point scale to estimate portion size; and (5) step five: manual calculation via equation to identify dietary fibre intake.


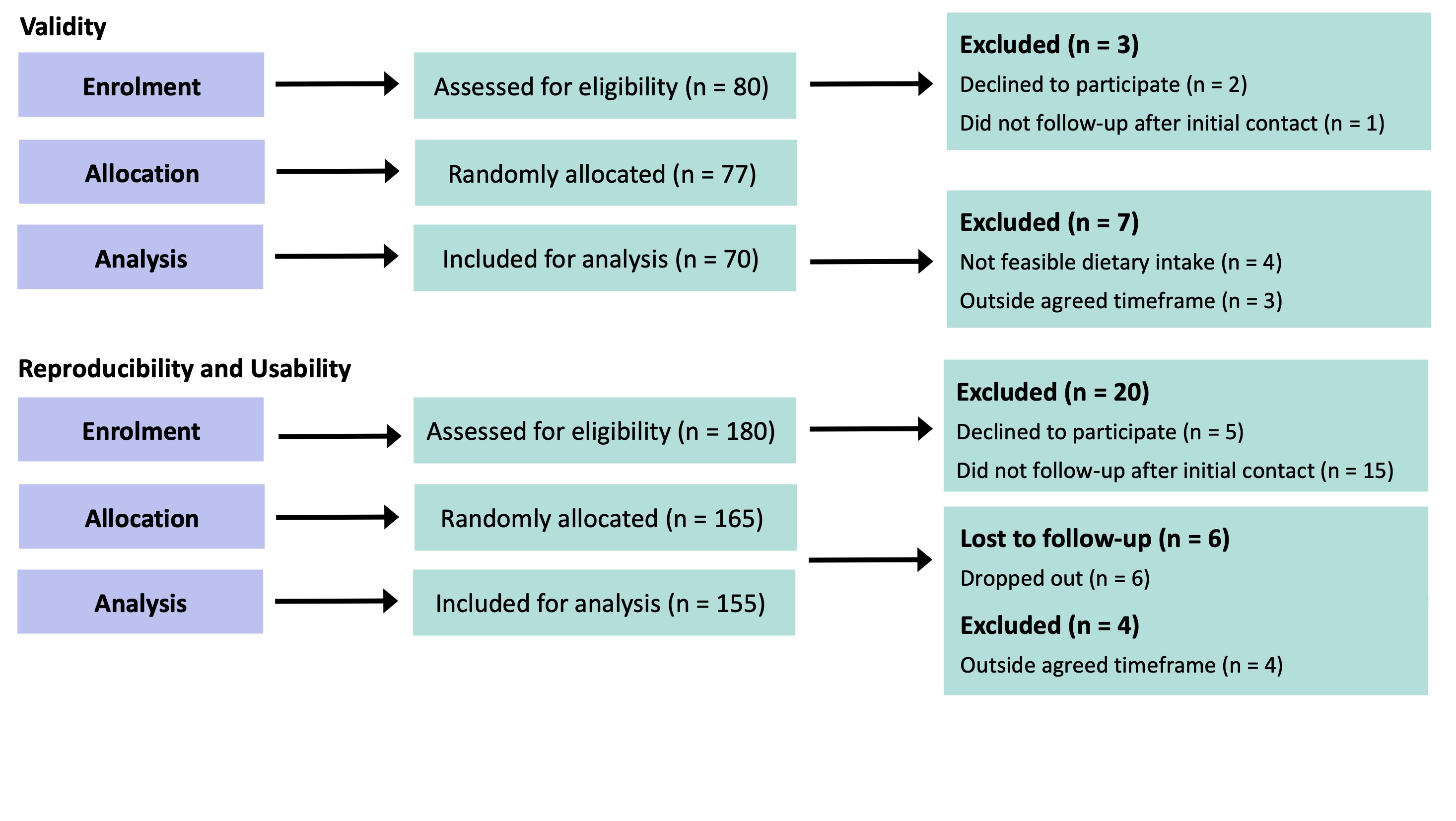
**Figure S3.** SCREEN-IT flowchart of participants.

**Table S2.** Age-derived reproducibility statistics with relevant interpretation (n = 155; 18-40 [n = 52]; 41-64 [n = 52]; and 65+ [n = 51]).

| **Dietary fibre (g/d)** | **ICC^1^** | **% difference^2^** | **Quartile agreement^3^** | **Weighted kappa^4^** |
| --- | --- | --- | --- | --- |
| 18-40 years | 0.94 | 1.44 | 44.2 | 0.26 |
| Interpretation* | Good | Good | Poor | Acceptable |
| 41-64 years | 0.78 | 0.65 | 50.0 | 0.33 |
| Interpretation* | Good | Good | Good | Acceptable |
| 65+ years | 0.86 | 0.18 | 55.8 | 0.42 |
| Interpretation* | Good | Good | Good | Acceptable |

*interpretation criteria as described by Lombard et al. [29]:

^1^Intra-class correlation coefficient (ICC) [good: >0.61, acceptable: 0.20-0.59, poor: <0.20]

^2^% difference [good: 0.0-10.9%, acceptable: 11.0-20.0%, poor: >20.0%]

^3^Cross-classification [18-40 – exact: 44.2%, adjacent: 38.5%, extreme: 17.3%; 41-64 – exact: 50.0%, adjacent: 32.7%, extreme: 17.3%; and 65+ – exact: 55.8%, adjacent: 34.6%, extreme: 7.7%]

^4^Weighted kappa [good: >0.61, acceptable: 0.20-0.59, poor: <0.20]

**
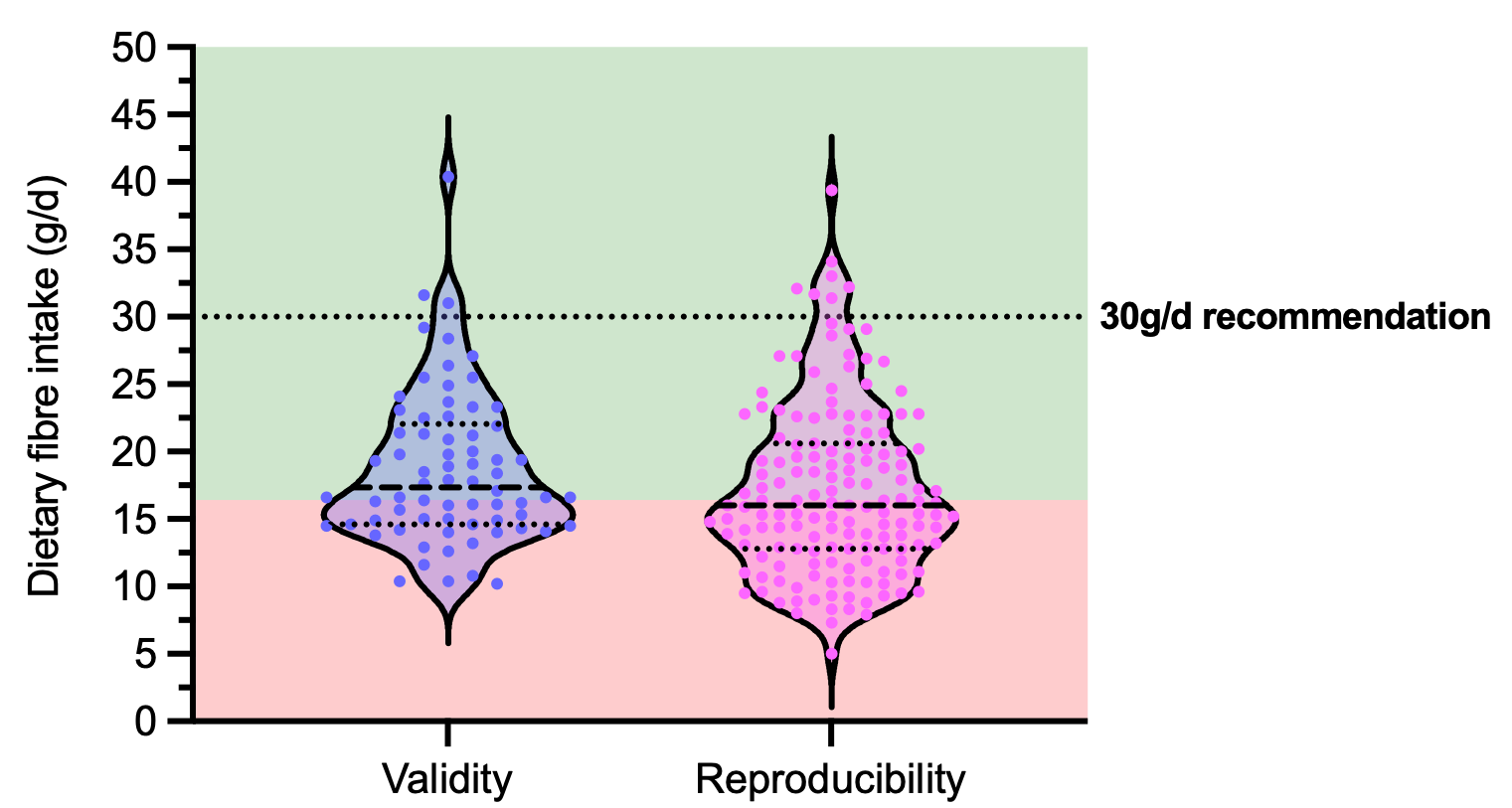
**

**Figure S4.** SCREEN-IT data visualisation (median: dashed line and quartiles: dotted lines) from validity (n = 70) and reproducibility (n = 155; combined intake from two occasions). Green and red shading (respectively) denote above or below National Diet and Nutrition Survey (NDNS) mean dietary fibre intake.
